# Metformin in Chemoprevention of Lung Cancer: A Retrospective Population-Based Cohort Study in Lithuania

**DOI:** 10.3390/medicina60081275

**Published:** 2024-08-07

**Authors:** Justinas Jonusas, Ausvydas Patasius, Mingaile Drevinskaite, Adomas Ladukas, Donata Linkeviciute-Ulinskiene, Lina Zabuliene, Giedre Smailyte

**Affiliations:** 1 Laboratory of Cancer Epidemiology, National Cancer Institute, LT-08406 Vilnius, Lithuania; 2 Department of Brachytherapy, National Cancer Institute, LT-08406 Vilnius, Lithuania; 3 Department of Public Health, Institute of Health Sciences, Faculty of Medicine, Vilnius University, LT-03101 Vilnius, Lithuania; 4 Medicine Clinic at Region Västmanland’s Hospital Västerås, Sigtunagatan, 721 89 Västerås, Sweden; 5 Institute of Clinical Medicine, Faculty of Medicine, Vilnius University, LT-03101 Vilnius, Lithuania

**Keywords:** type 2 diabetes mellitus, metformin, pioglitazone, lung cancer, chemoprevention, dose–response relationship

## Abstract

*Background and Objectives*: This study aimed to evaluate the potential chemopreventive effect of antidiabetic medications, specifically metformin and pioglitazone, on lung cancer in patients with type 2 diabetes mellitus (T2DM). Additionally, the potential dose–response relationship for metformin use was analyzed. *Methods*: We conducted a retrospective cohort study utilizing comprehensive national health insurance and cancer registry databases to gather a large cohort of T2DM patients. Cox proportional hazards regression models were used to assess the risk of lung cancer across different antidiabetic medication groups, adjusting for potential confounders such as age and gender. A dose–response analysis was conducted for metformin users. *Results*: Our results indicated that metformin users had a significantly lower lung cancer risk than the reference group (HR = 0.69, 95% CI [0.55–0.86], *p* = 0.001). The risk reduction increased with higher cumulative metformin doses: a metformin cumulative dose between 1,370,000 and 2,976,000 had an HR of 0.61 (95% CI [0.49–0.75], *p* < 0.001) vs. cumulative metformin dose >2,976,000 which had an HR of 0.35 (95% CI [0.21–0.59], *p* < 0.001). No significant association between pioglitazone use and the risk of lung cancer was found (HR = 1.00, 95% CI [0.25–4.02]). *Conclusions*: This study shows that metformin may have a dose-dependent chemopreventive effect against lung cancer in T2DM, while the impact of pioglitazone remains unclear and requires further investigation.

## 1. Introduction

Type 2 diabetes mellitus (T2DM) affects millions worldwide and has become a significant global health issue. Moreover, it is being recognized not only for its direct metabolic consequences but also as a potentially modifiable risk factor for several types of cancer, including lung cancer [1]. As one of the most prevalent cancers, lung cancer contributes significantly to global cancer-related morbidity and mortality rates [2]. Despite advancements in diagnostic and therapeutic guidelines, the overall survival rates remain relatively low, highlighting the need for effective preventive, screening, and early detection strategies [3].

Metformin, a first-line oral antidiabetic medication for T2DM, has been under investigation for its potential anticancer properties [4]. Accumulating evidence suggests that metformin acts as an anticancer agent through multiple mechanisms, such as activating AMP-activated protein kinase (AMPK), inhibiting the mammalian target of rapamycin (mTOR) signaling pathway, and suppressing the gluconeogenesis in the liver, thus decreasing the insulin levels and reduced mitogenic signaling [5,6]. Moreover, metformin has been shown to possess anti-inflammatory properties, which could further contribute to its chemopreventive potential [7]. However, published studies analyzing the association between metformin use and the risk of lung cancer have mixed results, with two large case–control studies reporting no protective effect of metformin in either men or women [8,9]. A study conducted in Switzerland, analyzing 13,043 diabetic patients with incident lung cancer, found that the long-term use of metformin (≥40 prescriptions) did not alter the risk of lung cancer (adjusted odds ratio (OR) = 1.09, 95% confidence interval (CI) [0.85–1.38]) [8]. Similarly, a Canadian cohort study of 115,923 new users of oral hypoglycemic agents, with 1061 lung cancer cases, showed no reduced lung cancer rate with metformin use (rate ratio 0.94, 95% CI [0.76–1.17]). No dose–response relationship was observed based on the number of prescriptions, cumulative duration, or dose [9]. On the other hand, a randomized controlled trial conducted in Australia and the USA among community-dwelling older adults with diabetes found that metformin use was associated with a reduced cancer incidence risk (adjusted hazard ratio (HR) = 0.68, 95% CI [0.51 to 0.90]), but no conclusive benefit for cancer mortality (adjusted HR = 0.72, 95% CI [0.43 to 1.19]) [10]. A meta-analysis of four studies conducted between 2009 and 2013 revealed that metformin therapy was associated with significantly lower risks of lung cancer (pooled relative risk (RR) = 0.71, with a 95% CI [0.55 to 0.95], *p* = 0.02), with evidence of moderate heterogeneity (I(2) > 50%) [11]. Another meta-analysis of 13 studies (10 cohort studies and 3 case–control studies published until September 20, 2017) showed that compared to non-metformin users, metformin probably decreased lung cancer incidence in diabetic patients (RR = 0.89; 95% CI [0.83–0.96]; *p* = 0.002) with significant heterogeneity (Q = 35.47, I(2) = 66%, *p* = 0.0004) [12]. Finally, a meta-analysis of six studies up to October 2020 with 539 participants showed that metformin may improve survival among patients with concurrent small-cell lung cancer and diabetes [13]. Patients using metformin had significantly longer overall survival (OS) and disease-free survival (DFS) than those not using metformin (OS: HR = 0.72 95% CI [0.53–0.98], *p* = 0.04; DFS: HR = 0.59 95% CI [0.45–0.76], *p* < 0.0001). The studies included were not significantly different in the two analyses by heterogeneity test, and there was no obvious publication bias.

Additionally, the role of pioglitazone in the risk of lung cancer is not clear. Pioglitazone is an agonist of the peroxisome proliferator-activated receptor gamma (PPAR-γ), which is associated with the regulation of cellular differentiation, proliferation, and apoptosis. PPAR-γ activation may provide additional antineoplastic effects by inhibiting the growth of cancer cells, suppressing angiogenesis, and inducing apoptosis [14]. Pre-clinical studies have shown that pioglitazone can prevent both adenocarcinoma and squamous-cell carcinoma either as a single agent or in combination with inhaled steroids and metformin [15]. Researchers from Minnesota demonstrated that in a benzo[a]pyrene (B[a]P)-induced carcinogenesis model in A/J mice, oral metformin at doses ranging from 500 to 1000 mg/kg/d is a viable chemopreventive treatment, modulating chemoprevention and anti-inflammatory biomarkers in residual adenomas. Pioglitazone at 15 mg/kg/d is also a viable chemopreventive agent at early-stage interventions. The combination of metformin and pioglitazone performed equally to metformin alone and better than pioglitazone alone [16]. However, as with metformin, data from observational studies have shown conflicting results. A cohort study from the California Diabetes Registry did not find an association between the use of pioglitazone and a decreased risk of lung cancer [17]. In contrast, two other large observational studies have reported nearly a 30% reduction in the risk of lung cancer in thiazolidinedione-treated patients [18,19]. On the other hand, a meta-analysis of studies up to June 2011 suggested a significantly lower risk associated with thiazolidinedione, including five observational studies (HR = 0.91, 95% CI [0.84–0.98]) and a null association with pioglitazone when including two studies (HR = 0.95, 95% CI [0.88–1.02]) [20]. A study of 37,900 never-users and 37,900 ever-users in the matched-pair sample supported a protective effect of pioglitazone on the development of lung cancer in Taiwanese patients with T2DM after a cumulative dose of over 15,300 mg. The slightly elevated risk associated with lower cumulative doses of pioglitazone requires further in-depth investigation. Furthermore, pioglitazone may alleviate the risk of lung cancer regardless of the presence or absence of smoking-related diagnoses of COPD and/or tobacco abuse [21]. A randomized phase II trial conducted in the USA, including 92 patients, showed that while pioglitazone did not improve endobronchial histology in this high-risk cohort, specific lesions showed histologic improvement, and further study is needed to better characterize the response to dysplasia [22].

Given these considerations, we conducted a retrospective cohort study to evaluate the potential chemopreventive effect of antidiabetic medications (metformin and pioglitazone) in patients with T2DM. Additionally, our study aimed to identify the potential dose–response relationship among metformin users. By utilizing comprehensive national health insurance and cancer registry databases, we aimed to provide valuable information for future research in this field.

## 2. Materials and Methods

### 2.1. Study Design and Population

In the present study, we utilized the National Health Insurance Fund (NHIF) database to identify patients with diabetes mellitus. This comprehensive database includes demographic data, as well as records of primary and secondary healthcare, emergency and hospital admissions, and prescriptions for reimbursed medications.

To identify cancer cases, we employed a record linkage technique with the Lithuanian Cancer Registry (LCR). The LCR is a comprehensive, population-based cancer registry that has maintained records of personal and demographic information, as well as diagnostic data for individuals diagnosed with cancer in Lithuania since 1978.

This retrospective cohort study investigated the association between antidiabetic medications and the risk of lung cancer. The cohort comprised male and female patients with an initial diagnosis of type 2 diabetes mellitus (International Classification of Diseases Australian modification, ICD-10-AM code E11) recorded in the NHIF database between 1 January 2000 and 31 December 2012. To ensure accurate classification of type 2 diabetes, we included only patients aged 40 years or older. Additionally, we excluded patients with fewer than six prescriptions for any antidiabetic medications, as well as those with fewer than six prescriptions for metformin (18,214 patients) and pioglitazone (89 patients).

To determine the incidence of lung cancer within the cohort, we linked diabetes records to the Lithuanian National Cancer Registry using personal identification numbers assigned to all Lithuanian citizens, incorporating data up to and including 31 December 2017. We considered only the first primary lung cancer diagnoses, excluding patients diagnosed with other cancers (23,603 patients) from the study. Participants with a lung cancer diagnosis preceding or within one year after their diabetes diagnosis (233 patients) were also excluded. Additionally, patients observed for less than one year were excluded from the final cohort (993 patients).

### 2.2. Outcomes

The person-time of observation was calculated from the date of the initial recorded diabetes mellitus diagnosis in the NHIF database to the date of lung cancer diagnosis, emigration, or the end of the observation period (31 December 2017), whichever occurred first.

The final cohort consisted of 91,326 patients diagnosed with type 2 diabetes mellitus, including 58,141 women and 33,185 men.

Antidiabetic medications prescribed for diabetic patients were categorized according to the Anatomical Therapeutic Chemical (ATC) classification system as follows: (1) pioglitazone and other medication users (including nine users of pioglitazone exclusively), (2) pioglitazone and metformin users, (3) metformin-only users, (4) metformin and other medication users, and (5) all other medication users. The last group was used as the reference group in the analysis, as it comprised diabetic patients who had never used pioglitazone or metformin.

To assess the dose–response relationship, we computed the cumulative dose using data from the NHIF database. Patients who had never been prescribed metformin after cohort entry were designated as never-users. In contrast, ever-users were classified into three groups based on their cumulative metformin dosage. The dose–response relationship was analyzed in two distinct cohorts: metformin ever-users and metformin-only users.

### 2.3. Statistical Analysis

To evaluate the potential chemopreventive effect of antidiabetic medication use on the risk of lung cancer, we estimated hazard ratios (HR) using univariate and multivariate-adjusted Cox proportional hazards models, accounting for gender and age at diabetes diagnosis. Additionally, we conducted multivariate-adjusted Cox proportional hazards models, incorporating age and gender, to estimate the impact of insulin type on the risk of lung cancer.

All statistical analyses were performed using STATA 15 software (StataCorp. 2020. Stata Statistical Software: Release 15.1 College Station, TX, USA).

## 3. Results

Table 1 provides the characteristics of the study population across different antidiabetic medication groups, including the distribution of males and females, mean age at diagnosis, and mean follow-up time.

The univariate and multivariate Cox proportional hazards regression model estimates for the risk of lung cancer, adjusted for gender and age at diagnosis, are shown in Table 2. In the multivariate analysis, female patients exhibited a significantly lower risk of lung cancer compared to males (HR = 0.18, 95% CI [0.15–0.25], *p* < 0.001). Age at diagnosis was positively associated with the risk of lung cancer (HR = 1.05, 95% CI [1.04–1.06], *p* < 0.001). Furthermore, the antihyperglycemic medication groups demonstrated varying risks of lung cancer relative to the reference group (Other), with the pioglitazone and metformin group presenting a significantly reduced risk (HR = 0.54, 95% CI [0.23–0.48], *p* = 0.001), as well as the metformin-only (HR = 0.69, 95% CI [0.55–0.86], *p* = 0.001) and metformin and other medication groups (HR = 0.64, 95% CI [0.54–0.78], *p* < 0.001).

Table 3 shows the univariate and multivariate Cox proportional hazards regression model estimates for the risk of lung cancer according to metformin dose in two separate cohorts: metformin users and metformin-only users. In the cohort of metformin users, a higher cumulative metformin dose was associated with a significantly reduced risk of lung cancer (>2,976,000, HR = 0.40, 95% CI: 0.32–0.50, *p* < 0.001). The same trend was observed among metformin-only users, showing a significantly lower risk of lung cancer in the highest cumulative metformin dose group (>2,976,000, HR = 0.35, 95% CI: 0.21–0.59, *p* < 0.001).

## 4. Discussion

### 4.1. Main Findings of the Study

In this retrospective cohort study, we investigated the potential chemopreventive effect of metformin and pioglitazone against lung cancer in patients diagnosed with T2DM. Our results show that metformin may possess chemopreventive properties (HR 0.69, 95% CI [0.55–0.86], *p* = 0.001). Additionally, a dose–response relationship was observed among metformin users, indicating that higher cumulative doses of metformin could be associated with a reduced risk of lung cancer. This was evident in both cohorts of metformin users and metformin-only users (HR = 0.40, 95% CI [0.32–0.50] vs. HR = 0.35, 95% CI [0.21–0.59], *p* < 0.001).

Our study demonstrated a significant possible chemopreventive effect associated with metformin use, which is consistent with previous research highlighting the anticancer properties of this antidiabetic drug.

### 4.2. Comparison with Previous Studies

In a study by Tsai et al., metformin usage was associated with a reduced risk of lung cancer (HR = 0.55, 95% CI [0.39–0.76], *p* < 0.001) [23]. Additionally, the authors demonstrated a dose–response relationship, with a greater effect observed among patients with higher cumulative metformin doses [23]. This chemopreventive effect is further supported by a large-scale Korean cohort study that included 732,199 subjects, reporting over a 50% decrease in lung cancer incidence among metformin users with higher cumulative doses (HR = 0.44, 95% CI [0.29–0.66], *p* = 0.007) [24]. Lastly, the protective role of metformin against lung cancer was reinforced by Xiao et al. in a meta-analysis comprising eighteen studies [25]. The authors showed a significant reduction in the risk of lung cancer (HR = 0.78, 95% CI [0.70–0.86]) associated with metformin usage. The researchers also observed a significant improvement in survival rates among lung cancer patients who used metformin (HR = 0.65, 95% CI [0.55–0.77]) [25]. Moreover, a recent meta-analysis, which included eight studies by Duan et al., showed that metformin may also have a positive potential effect on non-diabetic patients [26]. Authors reported that adjunctive metformin therapy may increase the disease control rate for patients diagnosed with advanced non-small-cell lung cancer (NSCLC) with an OR of 3.07 (95% CI [1.28–7.36], *p* = 0.01). However, there was no statistically significant difference between metformin users and control groups in progression-free survival (HR = 0.95, 95% CI [0.66–1.36], *p* = 0.77), overall survival (HR = 0.89, 95% CI [0.61–1.30], *p* = 0.55), or one-year progression-free survival (OR = 0.87, 95% CI [0.39–1.94], *p* = 0.73) [26]. Similar results were published in a meta-analysis by Wang et al., which included 19 studies analyzing patients diagnosed with NSCLC [27]. Even though pooled results showed that there was a positive effect on overall survival in the group of patients receiving metformin (HR = 0.84, 95% CI [0.71–0.98], *p* = 0.029), subgroup analysis indicated that metformin usage was beneficial only for patients diagnosed with diabetes (HR = 0.74, 95% CI [0.62–0.88] vs. HR = 1.00, 95% CI [0.60–1.67]) [27].

Recent data from pre-clinical and clinical studies suggest that metformin may have additional benefits beyond its glucose-lowering effects, including potential anticancer properties, including the activation of AMPK, the inhibition of the mammalian target of rapamycin (mTOR) signaling pathway, direct effects on cancer cells, the inhibition of gluconeogenesis, a reduction in insulin levels, a reduction in inflammation, and the modulation of the immune system. The activation of AMPK, a cellular energy sensor responsible for regulating cellular metabolism and maintaining energy homeostasis, is one of the most important anticancer mechanisms [28]. AMPK activation is linked to cell growth and proliferation inhibition and leads to pyroptosis via the AMPK-SIRT1-NF-κB pathway [29]. Metformin has been shown to significantly upregulate miR-7 via the AMPK pathway in a time- and dose-dependent manner. Additionally, the upregulation of miR-7 reduces non-small-cell lung cancer cell growth, migration, and invasion [30]. Metformin has also been found to inhibit the mammalian target of the rapamycin (mTOR) signaling pathway, which regulates cell growth, proliferation, and survival in some cancers, thus stopping tumor growth and progression [31,32]. Another notable anticancer mechanism of metformin is the suppression of gluconeogenesis in the liver in an LKB1- and AMPK-independent manner [33]. This lowers circulating glucose levels, reducing the availability of energy sources for cancer cells—a phenomenon known as the Warburg effect [34]. Furthermore, metformin exhibits anti-inflammatory and immunomodulatory properties that may contribute to its chemopreventive potential, such as the inhibition of pro-inflammatory cytokine production [35,36]. By reducing chronic inflammation, a well-established risk factor for cancer development, metformin helps in preventing cancer [37]. Moreover, metformin has been shown to modulate the immune system by promoting memory CD8+ T cell formation, enhancing their antiapoptotic abilities, and potentially supporting long-lasting cytotoxic functions in lung cancer patients [38]. Additionally, metformin reduces reactive oxygen species production, oxidative stress, and DNA damage by inhibiting mitochondrial complex I, thus reducing the risk of mutations [39]. Metformin has been shown to affect epigenetic modifications, particularly through its effects on DNA methylation and histone modifications, and may have potential as a promising therapeutic intervention with important implications for disease management and prevention [40]. Finally, combination therapies and targeted delivery strategies hold promise for improving the efficacy of metformin and reducing its adverse effects in cancer treatment and prevention [41,42,43,44,45]. Thus, long-term, randomized prospective studies are needed to confirm the potential benefit of metformin.

In contrast to metformin, the role of pioglitazone as a chemopreventive agent remains less clear. Our study did not find a significant association between pioglitazone use and the risk of lung cancer (HR = 1.00, 95% CI [0.25–4.02]). However, it is essential to acknowledge that the number of pioglitazone users in our cohort was relatively small, limiting the statistical power to detect a potential effect. Pioglitazone, a PPAR-γ agonist, regulates various cellular processes, including differentiation, proliferation, and apoptosis [46,47,48], which are crucial for inhibiting cancer development and progression. Nevertheless, the relationship between pioglitazone and the risk of lung cancer is complex and remains a subject of discussion. A double-blind, randomized, phase II placebo-controlled trial by Keith et al. showed that participants receiving oral pioglitazone did not exhibit improvement in bronchial dysplasia during a six-month intervention (mean change in histological score: –0.15, 95% CI [–0.52–0.22], *p* = 0.412) [22]. Given the inconsistent findings and the limited sample size of pioglitazone users in our study, the impact of pioglitazone on the risk of lung cancer warrants further investigation in larger and more diverse patient populations. Future research should aim to elucidate the molecular mechanisms underlying the potential effects of pioglitazone on all types of cancer development, as well as considering potential confounding factors such as the duration of pioglitazone use, dosage, and others.

### 4.3. Clinical Implications

Clinicians should focus their efforts on raising awareness about the potential prevention of lung cancer, including the avoidance of modifiable risk factors, early screening, and chemoprophylaxis, especially in patients at higher risk of lung cancer. Our study highlights the potential dual benefit of metformin for its blood glucose-lowering effect and its long-term benefit for lung cancer prevention, considering the possible side effects of its use and patient-specific health factors. The study results showed a dose–response relationship, with higher cumulative doses of metformin being associated with a lower risk of lung cancer, so it is important to start early the treatment of diabetes with metformin in the absence of contraindications to metformin use. Secondly, the study found a significant difference in lung cancer risk between genders, with female patients having a lower risk compared to males. This gender difference should be considered both in clinical evaluation and in developing personalized treatment and prevention strategies for T2DM patients. Additionally, age at diagnosis was positively associated with the risk of lung cancer, indicating that older patients with T2DM are at higher risk and may benefit from more age-specific lung cancer risk management and screening. Our study highlights the need for personalized management approaches based on patient age, gender, and specific medication use, which are important in daily practice, potentially integrating cancer prevention strategies into diabetes treatment plans.

### 4.4. Strengths and Limitations

Several strengths of our study should be mentioned. First, we used comprehensive national health insurance and cancer registry databases, which allowed us to assemble a large cohort of T2DM patients with detailed information on antidiabetic medication use and cancer outcomes. This reliable data source increases the generalizability of our findings and reduces the possibility of selection bias.

Our study has certain limitations. Firstly, as a retrospective study, our study contains inherent limitations of observational studies, including potential residual confounding. Secondly, we were unable to account for several important risk factors for lung cancer, such as smoking status, occupational exposure, and a family history of cancer. Future research, including a sensitivity analysis of these variables, should be conducted to take into account these potential confounding factors in order to understand further the relationship between antidiabetic medication use and the risk of lung cancer. Additionally, the relatively small number of pioglitazone users in our cohort may have limited the statistical power of our study to detect a significant association between pioglitazone use and the risk of lung cancer. Future studies with larger cohorts are needed to definitively establish the role of pioglitazone in lung cancer prevention.

## 5. Conclusions

In conclusion, our study shows that metformin may have a dose-dependent chemopreventive effect against lung cancer in T2DM, while the impact of pioglitazone remains unclear and requires further investigation.

## Figures and Tables

**Table 1:** Characteristics of the study population.

Antidiabetic Medication Group	Male, N	Female, N	Mean Age at Diagnosis, Years (SE)	Mean Follow-Up Time, Years (SE)
Pioglitazone and other	68	86	60.5 (10.3)	12.0 (4.1)
Pioglitazone and metformin	2021	3925	56.1 (8.7)	13.1 (3.5)
Metformin only	9209	17,569	62.2 (10.3)	8.9 (3.6)
Metformin and other	15361	26,629	60.3 (9.9)	11.3 (4.1)
Other	6526	9932	66.7 (11.8)	9.1 (4.8)

N—number; SE—standard error.

**Table 2:** Univariate and multivariate Cox proportional hazards regression model estimates for lung cancer.

Variable	Univariate				Multivariate			
	HR	95% CI		*p*	HR	95% CI		*p*
**Gender**								
Male	1.00				1.00			
Female	0.22	0.18	0.26	<0.001	0.18	0.15	0.25	<0.001
**Age at diagnosis**	1.03	1.02	1.04	<0.001	1.05	1.04	1.06	<0.001
**Antihyperglycemic medication**								
Other	1.00				1.00			
Pioglitazone and other	0.83	0.21	3.34	0.79	1.00	0.25	4.02	0.99
Pioglitazone and metformin	0.32	0.23	0.48	<0.001	0.54	0.37	0.78	0.001
Metformin only	0.53	0.43	0.66	<0.001	0.69	0.55	0.86	0.001
Metformin and other	0.49	0.41	0.59	<0.001	0.64	0.54	0.78	<0.001

HR—Hazard ratio; CI—Confidence interval; *p*—significance.

**Table 3:** Cox proportional hazards regression model estimates for the risk of lung cancer in metformin and metformin-only user cohorts according to the cumulative metformin dose.

Dose	HR	95% CI		*p*
Metformin and other users				
<1,370,000	1.00			
1,370,000–2,976,000	0.61	0.49	0.75	<0.001
>2,976,000	0.40	0.32	0.50	<0.001
Metformin-only users				
<1,370,000	1.00			
1,370,000–2,976,000	0.84	0.60	1.19	0.33
>2,976,000	0.35	0.21	0.59	<0.001

HR—Hazard ratio; CI—Confidence interval; *p*—significance.

## Data Availability

The data presented in this study are available on request from the corresponding author.
